# Management of Hyperglycemia in Hospitalized Patients Receiving Parenteral Nutrition

**DOI:** 10.3389/fcdhc.2022.829412

**Published:** 2022-02-21

**Authors:** Katja A. Schönenberger, Emilie Reber, Christa Dürig, Annic Baumgartner, Andriana Efthymiou, Valentina V. Huwiler, Markus Laimer, Lia Bally, Zeno Stanga

**Affiliations:** ^1^ Department of Diabetes, Endocrinology, Nutritional Medicine and Metabolism, lnselspital, Bern University Hospital, University of Bern, Bern, Switzerland; ^2^ Division of Clinical Pharmacy and Epidemiology, Department of Pharmaceutical Sciences, University of Basel, Basel, Switzerland

**Keywords:** hyperglycemia, hypoglycemia, type 1 diabetes mellitus, type 2 diabetes mellitus, parenteral nutrition

## Abstract

Almost half of inpatients on parenteral nutrition experience hyperglycemia, which increases the risk of complications and mortality. The blood glucose target for hospitalized patients on parenteral nutrition is 7.8 to 10.0 mmol/L (140 to 180 mg/dL). For patients with diabetes, the same parenteral nutrition formulae as for patients without diabetes can be used, as long as blood glucose levels can be adequately controlled using insulin. Insulin can be delivered *via* the subcutaneous or intravenous route or, alternatively, added to parenteral nutrition admixtures. Combining parenteral with enteral and oral nutrition can improve glycemic control in patients with sufficient endogenous insulin stores. Intravenous insulin infusion is the preferred route of insulin delivery in critical care as doses can be rapidly adjusted to altered requirements. For stable patients, insulin can be added directly to the parenteral nutrition bag. If parenteral nutrition is infused continuously over 24 hours, the subcutaneous injection of a long-acting insulin combined with correctional bolus insulin may be adequate. The aim of this review is to give an overview of the management of parenteral nutrition-associated hyperglycemia in inpatients with diabetes.

## 1 Introduction

Intravenous (IV) infusion of nutrients, i.e. parenteral nutrition (PN), is a form of nutritional support indicated when the nutritional requirements cannot be met by oral intake or enteral nutrition (EN). PN usually contains glucose, lipids, and amino acids. Electrolytes, vitamins and trace elements are added to the PN bag or provided separately. Nowadays, all-in-one admixtures in three-chamber bags are widely available, otherwise the hospital pharmacy can compound PN according to patients’ individual needs (1). PN can be administered either continuously or cyclically (e.g. overnight) (2).

Diabetes is frequent among adult inpatients, with an estimated prevalence of around 20%, which is increasing with older age (3–6). Consequently, diabetes is also on the rise in patients who require nutritional support. Although overweight and obesity are highly prevalent among patients with type 2 diabetes mellitus (T2DM), malnutrition and weight loss during hospitalization are harmful in these patients since they are accompanied by major muscle loss and associated with adverse outcome (7–9). Therefore, nutritional therapy is indicated in all malnourished patients and in certain cases PN is indicated regardless of the initial body mass index.

Risks of PN include complications associated with the central venous catheter (septic and mechanical complications, thrombosis, catheter occlusion) as well as acute- and long-term metabolic complications (e.g. hyper-/hypoglycemia, hypertriglyceridemia, hypercapnia, refeeding syndrome, acid-base disturbances, liver complications, manganese toxicity, metabolic bone disease) (10, 11). Hyperglycemia is often induced by nutritional support, even in patients without a diagnosis of diabetes (stress hyperglycemia). Overall, more than half of all inpatients receiving PN experience hyperglycemia (12–14). Underlying diabetes and hyperglycemia before starting PN are predictors for PN-associated hyperglycemia (13, 14). Cheung et al. showed that the complication risk in patients receiving PN increases by a factor of 1.58 for each 1 mmol/L (18 mg/dL) increase in glycemia above 6.3 mmol/L (114 mg/dL) (15). In particular, hyperglycemia in patients receiving PN is associated with an increased risk of cardiac complications (OR 1.61), infection (OR 1.4), sepsis (OR 1.36), acute renal failure (1.47) and death (OR 1.77) (15). In addition, glucose is lost during hyperglycemia through glycosuria [renal threshold ~10 mmol/L (~180 mg/dL)], which may lead to energy loss and consequently to deterioration of the nutritional status (16). The renal threshold in patients with insulin resistance is at 10.5 mmol/l (189 mg/dL) higher than in patients without insulin resistance (17).

The indication for nutritional support in patients with diabetes is primarily due to concomitant diseases and follows the same principles of nutritional support as for patients without diabetes. However, patients with diabetes have a particularly high insulin need, due to the decreased insulin sensitivity during acute illness (inflammation) combined with the lack of insulin in patients with type 1 diabetes mellitus (T1DM) or insulin resistance in T2DM (18).

The same PN admixtures may be used for patients with and without diabetes with additional insulin administration. Alternatively, the PN composition may be adapted to facilitate blood glucose control. The insulin administration is not only crucial to control blood glucose, but a lack of insulin may also increase muscle catabolism during acute illness. As a beneficial side effect, adding insulin to PN results in more rapid correction of malnutrition (anabolic effect) while glucose should be administered as soon as blood glucose levels are in target range to prevent a catabolic metabolic state (19).

The aim of this review is to give an overview of the management of PN-associated hyperglycemia in inpatients with diabetes.

## 2 Blood Glucose Control in Patients on Parenteral Nutrition

Consensus statements among organizations of health care professionals involved in inpatient diabetes care (e.g. American Diabetes Association, The Endocrine Society) as well as artificial nutrition (e.g. European Society of Clinical Nutrition and Metabolism [ESPEN], American Society for Parenteral and Enteral Nutrition [ASPEN]), recommend a blood glucose target of 7.8 to 10.0 mmol/L (140 to 180 mg/dL) for the majority of patients treated with insulin (20–23). During the first days of PN, frequent monitoring of glycemia is required and can be reduced when the patient has reached a stable metabolic state (18).

**Figure 1:**
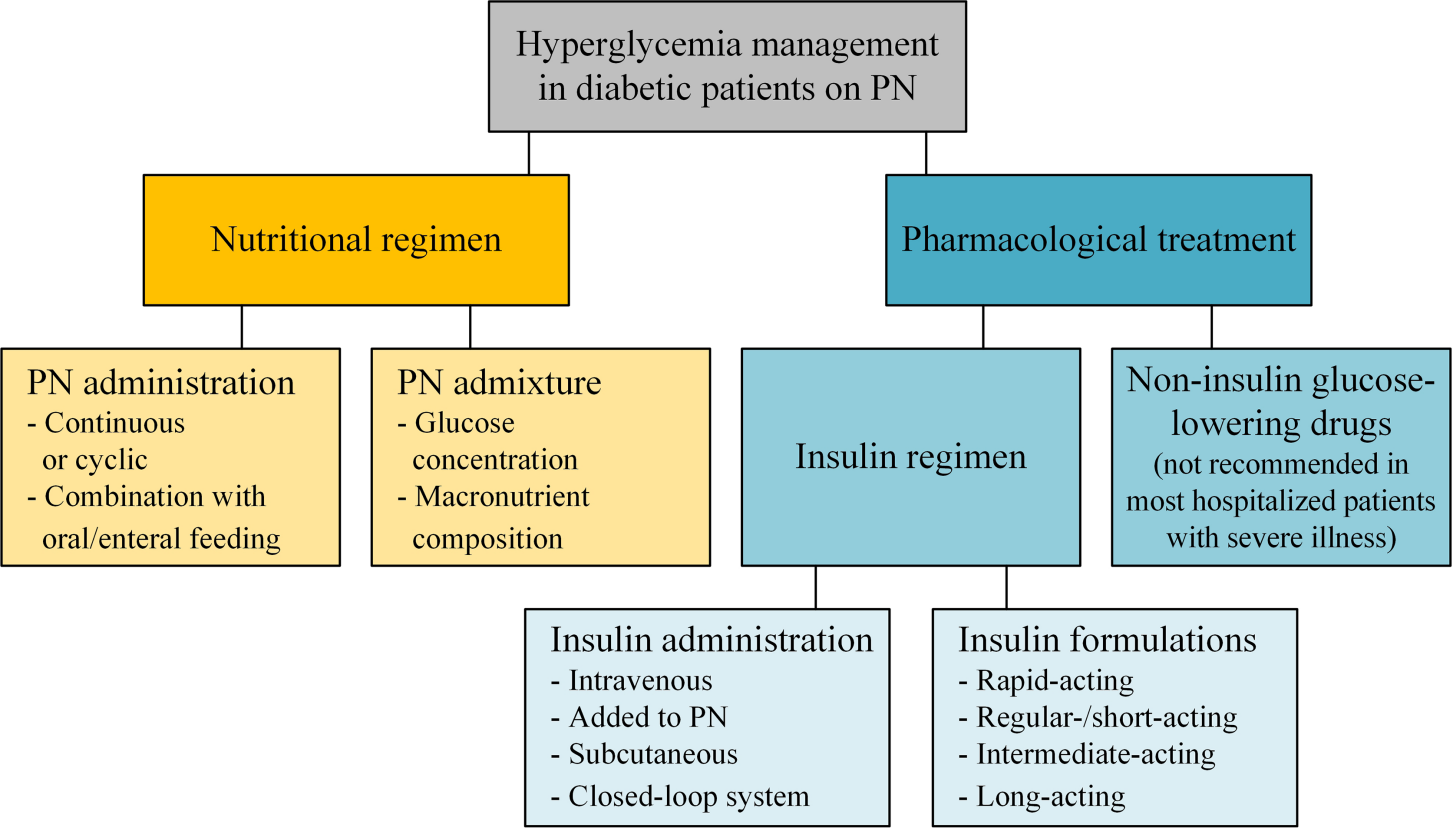
Hyperglycemia management of patients with diabetes receiving parenteral nutrition.

Both the nutritional and the insulin regimen need to be considered when controlling blood glucose in patients with diabetes receiving PN (**Figure 1**). While patients in the early stage of T2DM can be managed with non-insulin glucose lowering drugs, oral antidiabetic drugs in hospitalized patients are not recommended in most patients with severe illness. Moreover, evidence supporting the efficacy of oral antidiabetic drugs to reduce hyperglycemia in response to PN is very weak and most patients with diabetes require supplemental insulin when glucose is infused (18). Therefore, this review focuses on the nutritional and insulin regimen during PN.

### 2.1 Nutritional Regimen

Nutritional strategies can improve blood glucose control in patients receiving PN. Adaptations of the nutritional regimen include combining PN with oral intake and/or EN, reducing energy administration, reducing glucose content of the PN, or using non-glucose carbohydrate sources in PN (24). While these strategies have been studied in critically ill patients with insulin resistance, it is often unclear how these findings can be translated to non-critically ill patients with diabetes.

PN should be combined with oral intake and/or EN whenever possible as it improves intestinal integrity and stimulates incretin production. Oral glucose has a greater stimulatory effect on insulin secretion than IV glucose due to the incretin effect, mediated by the secretion of glucose-dependent insulinotropic peptide (GIP) and glucagon-like peptide 1 (GLP-1) as well as other gastrointestinal peptides (25). The combination of PN with oral intake and/or EN can thus contribute to improved blood glucose control. In post-operative patients with diabetes, the combination of PN with EN leads to reduced blood glucose concentrations, reduced insulin resistance, increased GIP and improved intestinal permeability compared to PN alone (26, 27). However, a combination of PN with oral intake or EN is not possible in certain clinical situations and the whole nutritional requirements need to be covered by PN (total PN). To determine the energy needs, the ESPEN recommends an indirect calorimetry measurement or an estimation with prediction equations, e.g. Harris-Benedict (28), or a weight-based formula, e.g. 25-30 kcal/kg body weight/day (29).

Reducing the energy supply can lead to a better blood glucose control and thus reduce the risk of hyperglycemia (24). In critically ill patients, hypocaloric PN (≤20 kcal/kg body weight/day or ≤80% of the estimated energy requirements) with an appropriate amount of proteins (≥1.2 g/kg body weight/day) is indicated during the first seven days as it may reduce the potential for hyperglycemia and insulin resistance (30). There is no evidence for whether these recommendations can be transferred to non-critically ill patients with diabetes while preventing malnutrition and its related negative clinical outcomes. In clinical practice, it is thus necessary to evaluate if a short-term energy deficit is tolerable or if longer-term PN is required. Generally, moderate amounts of glucose should be administered initially in patients with diabetes. As soon as the patient is in a stable metabolic condition, the PN administration should be increased to total requirements to prevent catabolism. In clinical practice, PN is often administered in a gradually increasing mode to improve metabolic tolerance and to reduce the risk of refeeding syndrome (31). Consequently, a dietician or other qualified and experienced health care professional in collaboration with the attending physicians should carefully assess patients’ energy requirements and nutritional status to develop an appropriate PN administration plan (29).

Besides reducing the absolute PN administration, adapting the macronutrient distribution may be a potential approach to avoid unfavorable consequences of excess glucose while covering the complete energy requirements. This is especially useful if PN is required long-term and a calorie deficit is not acceptable. When compounding PN, the hospital pharmacy can increase the fat content and decrease the glucose part to obtain an isocaloric admixture with reduced glucose. In critically ill patients, reducing the glucose content in PN to 150-200 g/day may be reasonable to prevent hyperglycemia (32, 33). Moreover, lipid-based PN (PN admixture containing 15% of energy as glucose, 15% as amino acids, and 70% as triglycerides) leads to a lower increase in blood glucose and insulin levels in this population (34). Olive oil-based lipid emulsions (80% refined olive oil in the lipid emulsion) are also associated with lower blood glucose levels (35). Despite a lack of evidence for the benefit and safety of isocaloric, glucose reduced PN in non-critically ill patients with hyperglycemia (27), it may be considered as a strategy for optimal blood glucose control in patients with diabetes.

Replacing glucose in PN by fructose or sorbitol is not recommended in patients with diabetes because these substrates do not improve the metabolic situation and plasma levels of these substrates are not routinely measured. Despite the fact that fructose, glycerol, sorbitol and xylitol are metabolized rapidly and in the beginning independent of insulin and have a lower effect on blood glucose concentration compared to glucose infusion (36), there is no difference in glycemic control or in insulin demand between glucose and a glucose-fructose-xylitol mixture in patients with diabetes (37). Furthermore, glucose is a widely available and affordable carbohydrate substrate in PN and should be the first choice for patients with diabetes (37).

Finally, in critically ill and surgical patients, glutamine-supplemented PN (0.2–0.5 g/kg body weight/day glutamine dipeptide, primarily added to the PN bag) leads to reduced infectious complication rates, hospital length of stay, and potentially mortality (38–40). Glutamine also improves blood glucose profile through a positive effect on glucose oxidation and insulin resistance (41). Although no studies were conducted specifically on the effect of glutamine-supplemented PN in patients with diabetes, critically ill patients receiving glutamine-supplemented PN have less hyperglycemia and need insulin less frequently than patients receiving PN without glutamine (42–44).

A *post-hoc* analysis of the Insulin in Parenteral Nutrition (INSUPAR) trial investigated the effect of PN with fish oil emulsions rich in ω-3 polyunsaturated fatty acids (PUFA) in T2DM (45). While patients with fish oil enriched PN had significantly lower triglyceride levels compared with other lipid emulsions, there were no differences in mean capillary glucose, glycemic variability, and insulin dose. However, there were significantly more hypoglycemic events in the fish oil group, which might have been caused by an overrepresentation of patients with end-organ damage and significantly longer PN duration in the fish oil group. Therefore, further research to examine the effect of ω-3 PUFA in patients with diabetes receiving PN is needed (45).

### 2.2 Insulin Regimen

The preferred method to manage blood glucose in patients with diabetes receiving PN is to administer a rapid-acting insulin IV. As this is rarely possible in general wards, a regular/short acting insulin can alternatively be added to the PN bag. Other options in stable patients are subcutaneous injection of long-acting insulin combined with short-acting insulin to cover additional prandial carbohydrate intake (in case of a combination of PN and enteral/oral intake) or the use of a subcutaneous insulin pump. Moreover, any combination of the above may be used and practice varies widely among patient populations, disciplines and individual clinicians. The advantages and particularities of these options are summarized below.

#### 2.2.1 Intravenous Insulin Infusion

**Figure 2:**
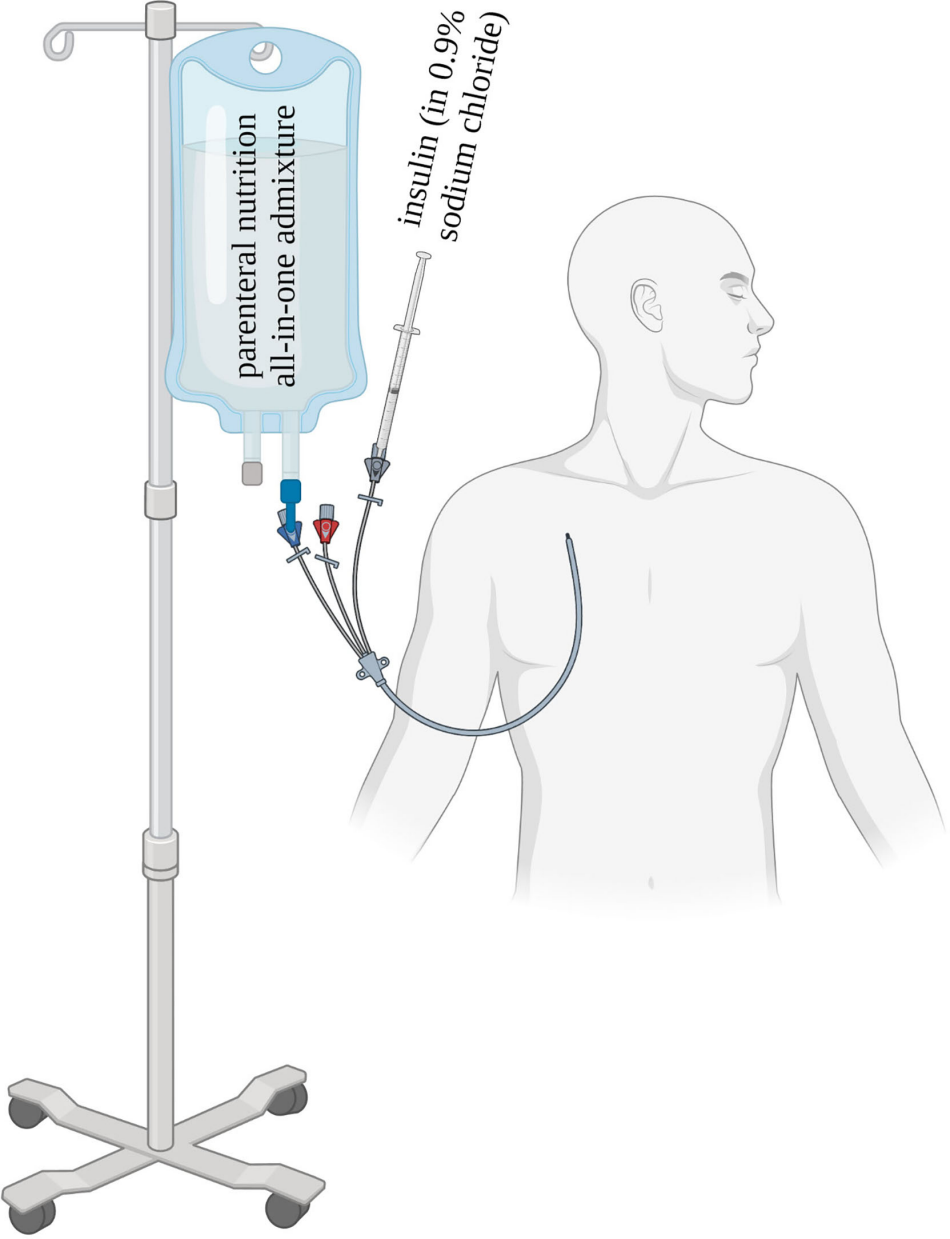
Parenteral nutrition and insulin infusion in unstable patients with diabetes [adapted from (46)].

Ideally, insulin is administered continuously with an IV infusion in patients receiving PN (**Figure 2**) due to favorable pharmacokinetics in these patients. This is especially important for unstable and critically ill patients where maximal flexibility of the management is needed in order to allow imminent adaptation to rapidly changing circumstances, yet without increasing the risk of hypoglycemia. In critically ill patients, higher levels of glucagon, cortisol and catecholamines further favor hyperglycemia and insulin resistance (47, 48). In order to achieve stable glycemia within the defined target range, frequent adaptations of the insulin infusion rate are recommended. The application of IV insulin boluses may lead to serious glycemic fluctuations and provoke dyselectrolytemia, in particular hypokalemia (18). As a starting point, the insulin dose in **Table 1** can be used and blood glucose levels should be checked every two hours. Woolfson et al. (49) published an algorithm for the dynamic insulin administration during PN according to the current blood glucose and the direction of change since the last measurement (**Table 2**).

**Table 1:** Initial intravenous insulin dose during parenteral nutrition.

Blood glucose [mmol/L]	Blood glucose [mg/dL]	Intravenous insulin [IU/h]
6.0-7.9	108-143	1
8.0-9.9	144-179	2
10.0-11.9	180-215	3
12.0-13.9	216-251	4
14.0-15.9	252-187	5
16.0-17.9	288-323	6
18.0-19.9	324-359	7
≥20.0	≥360	8

**Table 2:** Dynamic insulin administration according to the current blood glucose and the direction of change since the previous measurement [according to (49)].

Blood glucose		Insulin infusion rate [mL/h]	Insulin concentration
**[mmol/L]**	**[mg/dL]**		
<4.0	<72	Decrease by 1.0	If rate becomes ≤0.5 mL/h: Halve concentration and restart at 0.5 mL/h
4.0-6.9	72-125	Decrease by 0.5	
7.0-10.9	126-197	Same rate	
11.0-15.0	198-270	If lower than previous test: Same rate	If rate becomes ≥4.5 mL/h: Double concentration and restart at 2.5 mL/h
		If higher than previous test: Increase by 0.5	
>15.0	>270	If lower than previous test: Same rate	
		If higher than previous test: Increase by 1.0	

It is recommended to indicate on the PN bag that insulin is running on a separate IV infusion in order to check the insulin application when PN is paused or stopped. In patients with T1DM, a minimal infusion rate of 0.5-1.0 IU/h should always be maintained as a basal rate, independent from the PN administration.

#### 2.2.2 Insulin Added to Parenteral Nutrition

Adding insulin to PN is a convenient and physiologically favorable method to treat hyperglycemia. However, manipulating a PN bag increases the risk of infectious complications. Nevertheless, the hospital pharmacy may add insulin to PN bags or, if the hospital pharmacy allows, the insulin may be added to PN bags directly on the ward. This form of insulin delivery is particularly advantageous for cyclic administration of PN or when PN needs to be stopped frequently, e.g. for examinations requiring a fasted state, since the insulin stops when the nutrition stops (21). Furthermore, it is useful in general wards, when separate IV insulin administration is not feasible. Nevertheless, it is inappropriate in unstable conditions since the frequency of dosage adjustment should not be more than every 24 hours and the insulin concentration cannot be adapted once it is in the PN bag.

**Figure 3:**
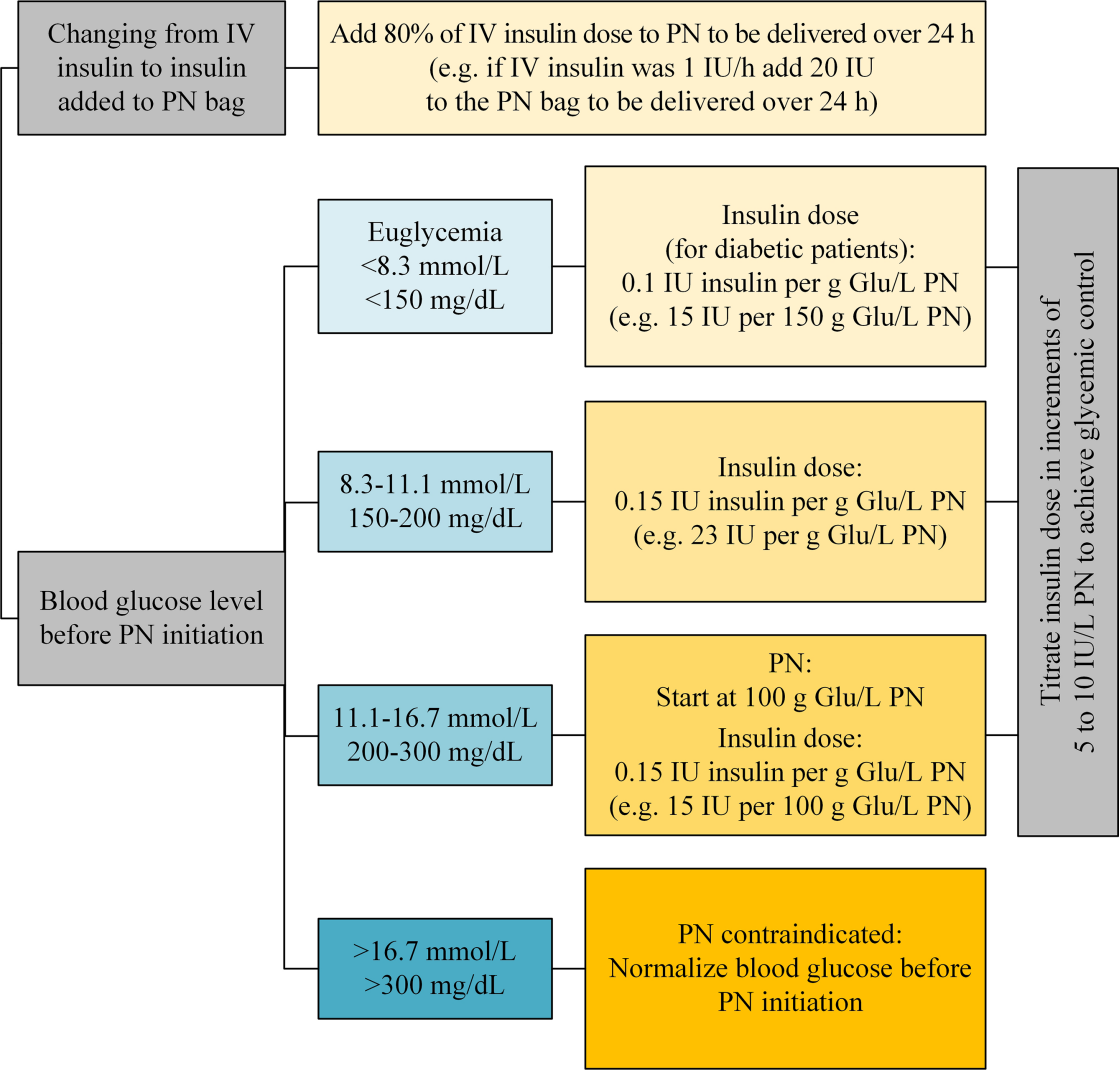
Insulin dose added to PN bag considering the glucose content of PN and the insulin resistance (Glu, glucose; IV, intravenous; PN, parenteral nutrition) (51–54).

Due to higher amounts of rapidly metabolized carbohydrate contents of PN in comparison to oral food, insulin needs are slightly higher than for oral nutrition (48). Obese patients with T2DM and significant insulin resistance may require as much as 1 IU of insulin for every 5 g of glucose while thin patients with T1DM may require only 1 IU for every 20 g of glucose (50). In patients switched from IV insulin to insulin added to the PN bag, insulin requirements can be estimated according to **Figure 3**. The amount of insulin can be titrated every one to two days, based on blood glucose monitoring. Since more frequent adjustments are impractical and costly, the concurrent use of rapid- or short-acting insulin as correction every six hours will help to fine-tune glycemic management (20, 21).

The randomized controlled INSUPAR trial compared 100% of regular insulin added to PN with 50% of regular insulin added to PN and 50% subcutaneous glargine insulin in non-critically ill T2DM patients (55). No differences were observed in the effectiveness to achieve an adequate metabolic control (venous blood glucose, capillary glucose, glycemic variability parameters, total insulin and capillary glucose decrements) during PN infusion. However, glycemic control after PN interruption was better in the group with glargine insulin. While no difference in the rate of hyperglycemia was observed, hypoglycemia was more frequent in the group with glargine insulin (55).

#### 2.2.3 Subcutaneous Insulin Injection

Post-acute, stable patients can be transitioned from IV to subcutaneous insulin administration. It is important to overlap IV and subcutaneous insulin delivery, starting subcutaneous administration two to three hours prior to IV discontinuation. The scheme in **Table 3** can serve as a rule of thumb and if in doubt, a diabetes specialist should be consulted. In particular within the first 24 hours after transition, additional glucose corrections may be necessary in order to achieve adequate glycemic control. If glycemic targets cannot be achieved within the first 48 hours, switching back to IV insulin infusions should be considered.

**Table 3:** Scheme for injection of subcutaneous insulin during parenteral nutrition.

Parenteral nutrition administration	Subcutaneous insulin injection
Continuous parenteral nutrition over 24 hours	Application of 100% of the insulin as long-acting insulin subcutaneously (basal insulin)
Continuous parenteral nutrition over 24 hours in combination with oral or enteral nutrition	Application of 50% of the insulin as long-acting insulin subcutaneously (basal insulin) and 50% as short-acting insulin subcutaneously
Cyclic parenteral nutrition over 12-14 hours overnight in combination with oral or enteral feeding	Application of 50% of the insulin as long-acting insulin subcutaneously (basal insulin) with emphasis on nocturnal coverage and 50% as short-acting insulin subcutaneously

In stable patients with diabetes receiving continuous PN, the subcutaneous injection of a long-acting insulin is useful (56). The use of ultra-long-acting insulin should be restricted to very stable situations due to limited flexibility. In case of oral intake in addition to PN, a long-acting insulin should be combined with short-acting insulin to cover prandial carbohydrate intake (51, 55) and, even if patients are on total PN, basal insulin should always be combined. Dosage of the short-acting insulin is based on preprandial blood glucose and expected carbohydrate intake. **Table 4** shows a scheme for insulin dosage adjustment based on preprandial blood glucose measurements and normal insulin sensitivity. Note that this scheme is a rule of thumb and the amount of insulin must be adapted according to glycemic control, dietary changes and insulin resistance.

**Table 4:** Scheme for injection of rapid-acting insulin for patients receiving parenteral nutrition (meal insulin, assuming oral or enteral intake of about 20 g carbohydrate and normal insulin sensitivity).

Preprandial blood glucose		Subcutaneous insulin [IU]
[mmol/L]	[mg/dL]	
6.0-7.9	108-143	2
8.0-9.9	144-179	4
10.0-11.9	180-215	6
12.0-13.9	216-251	8
14.0-15.9	252-187	10
16.0-17.9	288-323	12
≥19.0		14

This scheme is a rule of thumb, based only on preprandial blood glucose. The insulin dose must be adapted quickly depending on the blood glucose course and diet.

A combination of long-acting and short-acting insulin is also recommended in case of combined PN and EN, in particular in case of the application of EN by boluses. Of note, changes in the application of PN afford an instant adaptation of the insulin regimen in order to prevent hypoglycemia.

#### 2.2.4 Continuous Glucose Monitoring and (Hybrid) Closed-Loop Insulin Delivery

**Figure 4:**
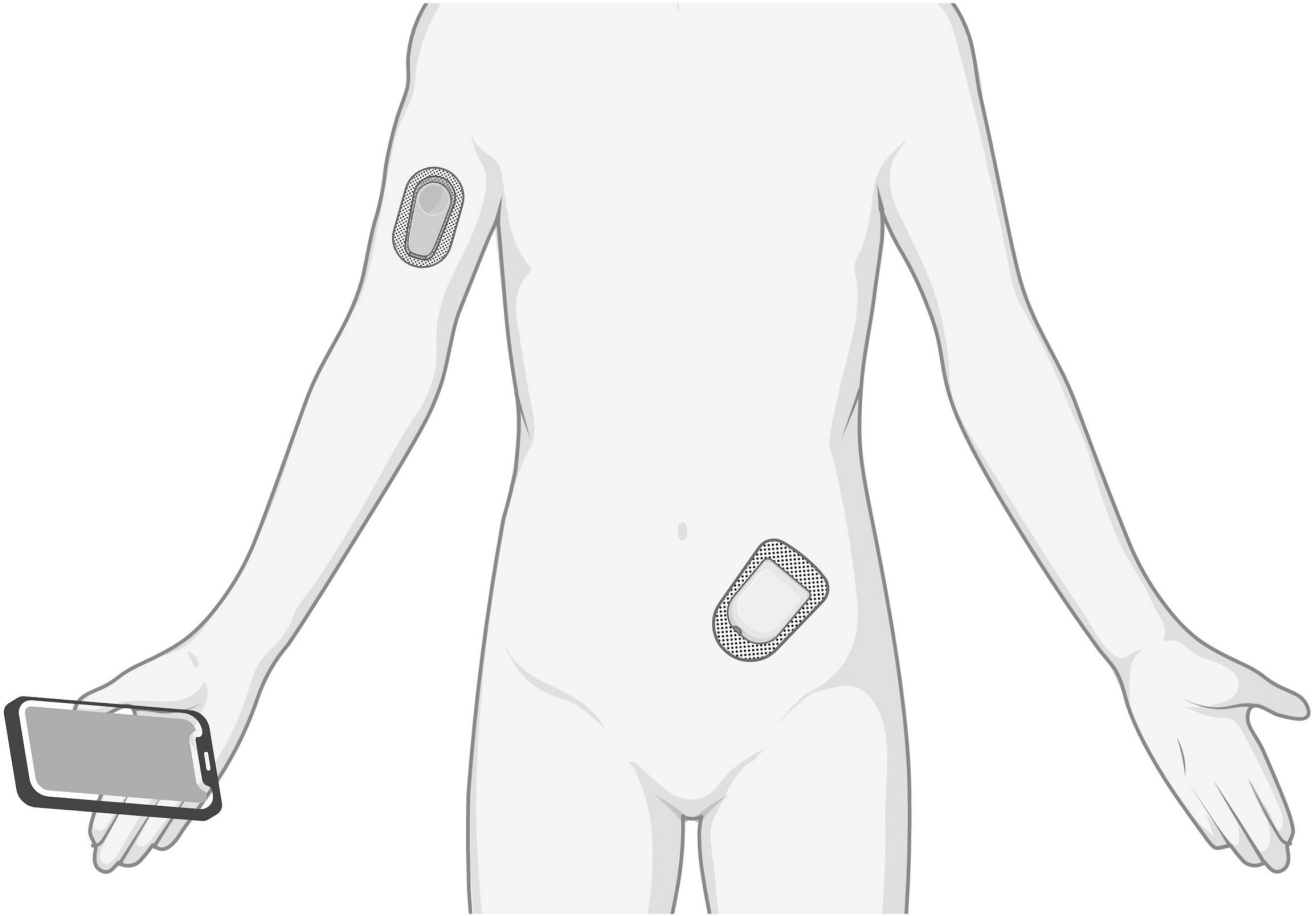
A continuous glucose monitor (arm) measures interstitial glucose levels and transmits them to a smartphone with a control algorithm. An insulin pump (abdomen) delivers a rapid-acting insulin.

Continuous glucose monitoring (CGM) devices have become the standard glucose monitoring tool for patients with diabetes (57). CGM measures interstitial glucose levels and can transmit them to a smartphone with a control algorithm to administer insulin subcutaneously *via* an insulin pump (**Figure 4**). This forms a hybrid closed-loop, i.e. automatic insulin administration for basal requirements and (depending of the type of insulin pump) automated glycemic corrections, or a fully closed-loop, i.e. with additional automated coverage of dietary carbohydrate intake or supply (58).

Patients with T1DM may be admitted to the hospital with a hybrid closed-loop system. Current guidelines of the American Diabetes Association (ADA) state that diabetes self-management may be appropriate for patients with adequate oral intake, who successfully self-manage their diabetes at home and have a good understanding of sick-day management (21). As people receiving PN do not have adequate oral intake, the use of a semi-closed loop system under PN needs to be closely supervised by a diabetologist. A further limitation is the delayed pharmacokinetics of the subcutaneous insulin administered *via* the insulin pump compared to IV administration.

Fully closed-loop systems are not yet available commercially, but can be used in research settings. If properly done, such a closed-loop system allows better blood glucose control than standard insulin therapy in hospitalized patients receiving nutritional support. In a randomized-controlled trial of 43 inpatients, the blood glucose levels were within the target range during eight additional hours daily in the closed-loop group, without increasing the risk of hypoglycemia (59). A study investigating continuous subcutaneous insulin infusion found significantly decreased glycemic variability and insulin requirements compared to a multiple daily injection regimen in patients with T2DM receiving PN after gastrointestinal surgery (60). Although some evidence about the efficacy of (hybrid) closed-loop systems in patients receiving PN exists, official recommendations are not available yet.

## 3 Compatibility, Stability and Availability of Insulin in Parenteral Nutrition Admixtures

Medication may be added to PN if (i) it is stable, (ii) it is compatible, (iii) evidence supports the clinical value, and (iv) the frequency of dosage adjustment is not more than 24 hours (61). Insulin is a potential candidate for admixture to PN. Insulin administration within the PN bag or *via* a Y-site is recommended by both International Societies for Clinical Nutrition, the ESPEN and ASPEN, if stability of both the PN admixture and the insulin have been tested previously (62, 63). However, unless compatibility is stated by the manufacturer, hospital pharmacists must always be consulted when insulin should be admixed directly into the PN admixture bag, since there may be many physicochemical issues associated with frequent harmful events (52).

There are only few data on the insulin stability in a PN admixture and they mainly refer to administration *via* Y-site. There is no data available regarding admixture of insulin analogues and it is impossible to extrapolate the results for different insulins and PN admixtures. Only regular/short-acting insulin is chemically stable in PN admixtures and may thus be added. Other formulations such as NPH, lispro, aspart or glargine are incompatible with PN admixtures. The maximum insulin concentration in PN admixture is 60 IU/L (52).

The stability and bioavailability of insulin admixed in PN ranges from 10% to 95% and primarily depends on the PN composition (mainly glucose content and pH), the insulin concentration as well as the contact time between PN admixture and insulin (64–67). The presence of vitamins and trace elements in the PN bag greatly influences the insulin recovery (available percentage of the initial insulin concentration): 95% in PN admixtures containing vitamins and trace elements versus 5% when they are not contained in PN (66, 68). However, the zinc contained as stabilizer for polypeptides in pharmaceutical solutions is also present in the trace elements solutions added in PN admixture bags and may cause insulin hexamerisation (67). Further chemical reactions may occur such as insulin glycation and interactions between insulin (or its binding components) and potential degradation products of the PN components (e.g. lipid peroxides).

Another relevant factor, the adsorption to the infusion set and PN bag may occur depending on the material and the PN flow rate. This is less of a problem with the newest bag material (ethylene vinyl acetate [EVA]) but remains a problem along the infusion line, especially at low flow rates (<2 mL/h) (69–71). The adsorption phenomenon is well documented but not well quantified. However, it reduces the expected and actual insulin delivery and is unaccounted for in therapy, contributing to high glycemic variability and poor control (72). Adsorption is not constant, e.g. insulin loss is highest in the first two hours of the use of new infusion lines, resulting in 20% to 80% delivery of the expected dose (66). Furthermore, adsorption increases as the flow rate decreases. A study of Knopp et al. showed that the adsorption capacity of the line doubled when the infusion flow rate was halved from 1 mL/h to 0.5 mL/h (72). This may have significant implications in some care settings, e.g. pediatric intensive care where it represents a loss of 30-60% of total insulin delivery over 24 hours (72). Some recommendations to reduce these losses have been made (insulin flushing, co-delivery of proteins) but did not find their place in clinical practice (wasting of insulin, time-consuming). Moreover, different analytical methods to measure insulin and different laboratory conditions (e.g. light exposure, temperature) may affect the results. In a study of Henry et al., total errors in insulin detection (sample concentration 5 to 40 IU/L) in a PN admixture with trace elements and vitamins varied between -0.32% and 8.37% for immunoradiometric assays and between 1.74% and 4.52% for immunoelectrochemiluminometric assay, the only one showing an accuracy profile within the accepted calculated tolerance limits (≤ ± 10%) (73). Separative methods require extraction steps, potentially modifying the proteins’ structure and hence altering the results (67). The reasons for these physicochemical effects are not fully understood to date (66). Considering that chemical reactions can cause conformational modification, decreasing the recognition, it is impossible to predict metabolic or clinical consequences. Thus, there is a need for clinical trials and other analytical tools (67).

Very few and heterogeneous studies, mostly excluding patients with diabetes, are available regarding the clinical effect of insulin added directly into PN bags. A recent meta-analysis comparing subcutaneous insulin glargine with regular insulin added to the PN bag did not show any differences in mean blood glucose levels or frequency of hypoglycemia nor on the reduction patterns of hyperglycemia or rates of hypoglycemia (74).

The currently scarce evidence and the clinical practice support the direct admixture of insulin in PN bags in stable inpatients, demonstrating good glycemic control and safety profile (66). However, insulin should only be added into PN bags once glycemia and PN regimen are stable, according to specific personalized protocols and under close blood glucose monitoring and surveillance of the clinical situation (66).

## 4 Interruption of Parenteral Nutrition

In general, PN can be discontinued without any reduction steps (62), e.g. in patients undergoing an intervention requiring be fasted. Rebound hypoglycemia when stopping PN with added insulin is very rare in adults (51). Nevertheless, PN is usually tapered off, since oral nutrition is built up gradually. Continuous insulin administration must be adapted when the PN is stopped.

When PN is interrupted, patients with T2DM should be followed with careful glucose monitoring. If hyperglycemia occurs, insulin should be administered. Patients with T1DM require overlapping insulin when PN is interrupted; otherwise hyperglycemia will develop if no insulin is administered and the shorter half-life of IV insulin increases the risk of ketosis (21). The amount and type of insulin depends on the expected duration of the interruption. Because of the possibility of accidental discontinuation of insulin administration when PN with insulin added to the bag is interrupted, some clinicians recommend that patients with T1DM receive a part of their basal insulin as an injection. This approach may also be useful for insulin dependent T2DM patients.

## 5 Conclusion

Hyperglycemia is frequent in patients receiving PN and inadequate blood glucose management as well as insulin administration increase the risk for adverse outcome. Blood glucose management of patients with diabetes receiving PN is complex and requires multiprofessional collaboration in a nutritional support team including physicians, diabetes specialists, pharmacists, dieticians, diabetes counseling and nurses. Establishing the blood glucose targets and control strategies necessitates interdisciplinary involvement while the responsibility for blood glucose control should be clearly defined and monitored. It is important to provide a defined protocol for insulin administration, so that it is carried out in a consistent manner by all health care personnel (52).

## Author Contributions

KS, ER, and ZS contributed to the conception. KS wrote the first draft of the manuscript. ER, CD, and AB wrote sections of the manuscript. ZS supervised the work. All authors contributed to manuscript revision, read, and approved the submitted version.

## Funding

This research was funded by the Division of Clinical Pharmacy and Epidemiology, University of Basel (third-party grant FO119900) and the Department of Diabetes, Endocrinology, Nutritional Medicine and Metabolism, Inselspital, Bern University Hospital (research fund WFE-002).

## Conflict of Interest

The authors declare that the research was conducted in the absence of any commercial or financial relationships that could be construed as a potential conflict of interest.

## Publisher’s Note

All claims expressed in this article are solely those of the authors and do not necessarily represent those of their affiliated organizations, or those of the publisher, the editors and the reviewers. Any product that may be evaluated in this article, or claim that may be made by its manufacturer, is not guaranteed or endorsed by the publisher.
